# Topical Collection: Natural Enemies and Biological Control of Plant Pests

**DOI:** 10.3390/insects13050421

**Published:** 2022-04-29

**Authors:** Eric Wellington Riddick

**Affiliations:** National Biological Control Laboratory, Agricultural Research Service, United States Department of Agriculture, Stoneville, MS 38776, USA; eric.riddick@usda.gov

Natural enemies have an extensive history as biological control agents against crop pests worldwide. Predatory insects and mites, parasitic wasps and flies, pathogenic bacteria, fungi, and viruses have been used against crop pests with varying degrees of success. This editorial aims to highlight articles that reveal recent advances or discoveries in fundamental and applied research on natural enemies and biological control.

One article tested the effects of UV-absorbing (photoselective) nets on the dispersion potential of a syrphid (*Sphaerophoria rueppellii* Wiedemnann) and its prey, aphids [1]. The authors concluded that the deployment of these nets and augmentative releases of *S*. *rueppellii* were compatible strategies in IPM aphid control programs [1]. In a review of the literature on volatile and non-volatile plant-derived organic compounds as potential oviposition stimulants for aphidophagous predators, the author indicated that volatile compounds were effective oviposition stimulants for syrphids (e.g., *Episyrphus balteatus* DeGeer) more so than for coccinellids or chrysopids [2]. Organic compounds with low-to-moderate molecular weights and moderate-to-high vapour pressures were more effective oviposition stimulants [2].

An article revealed complementary action of a generalist predatory mite *Anystis baccarum* (L.), family Anystidae, and augmentative releases of a hymenopterous aphid parasitoid *Aphidius ervi* Haliday, family Aphidiidae, to control the foxglove aphid *Aulacorthum solani* (Keltennbach) on sweet peppers cultivated in greenhouses [3]. The predatory mite became established in the greenhouse and benefited from attacking other insects such as western flower thrips *Frankliniella occidentalis* (Pergande), family Thripidae [3].

One article stressed the importance of rigorous morphological investigations to accurately identify predatory coccinellids (*Chilocorus* species) before release for classical biological control of coccids [4]. The authors found that *Chilocorus kuwanae* Silvestri and *Chilocorus renipustulatus* (Scriba) were the same species. Consequently, releases of *C*. *kuwanae* in Europe and the Caucasus did not represent classical biological control, since the same species (misidentified as *C*. *renipustulatus*) were native to these regions [4].

To facilitate cost-effective augmentative biological control, research has centered on mass production and artificial diet development for another coccinellid, i.e., *Stethorus gilvifrons*, a specialist predator on tetranychid spider mites [5]. A diet consisting of sucrose, honey, royal jelly, agar, yeast, date palm pollen supplemented with Mediterranean flour moth (*Ephestia kuehniella* Zeller) eggs and 2,4-dihydroxybenzoic acid was most effective and supported the development and reproduction of *S*. *gilvifrons* in the laboratory [5].

Mirid bugs can be effective predators of soft-bodied plant pests [6], but ongoing research is necessary to test the compatibility of mirids with other control technologies. In one study, the mirid *Nesidiocoris tenuis* (Reuter) was exposed to a plant-derived toxin, methyl benzoate (MB) in laboratory and greenhouse bioassays [7]. MB is a volatile organic compound with acute toxicity to aphids, whiteflies, and spider mites. A 1% MB concentration had little lethal or sublethal effect on *N*. *tenuis* adults [7]. This research suggested that MB and *N*. *tenuis* could be implemented together for integrated control of pests. In another article, researchers tested for side effects of ds-RNA technology on the mirid *N*. *tenuis* [8]. Results indicated that ds-RNA (RNA interference) designed to target the αCOP gene of a gelechiid moth *Tuta absoluta* (Meyrick) had no lethal or sublethal effects on *N*. *tenuis* in laboratory bioassays. The authors suggested that dsRNA and *N*. *tenuis* could be combined for integrated pest management of *T*. *absoluta* [8].

Other research tested the compatibility of two intraguild predators, a coccinellid *Cryptolaemus montrouzieri* (Mulsant) and a chrysopid *Chrysoperla carnea* (Stephens), and their potential to control mealybugs [9]. Intraguild predation was common between the two predators, usually in favor of *C*. *carnea*. However, the addition of extraguild prey, i.e., mealybug nymphs and *E*. *kuehniella* eggs, significantly reduced encounters between *C*. *montrouzieri* and *C*. *carnea* in laboratory arenas [9]. One article examined the predation potential of another coccinellid *Harmonia axyridis* (Pallas) and an anthocorid *Orius sauteri* (Poppius) against the noctuid *Spodoptera frugiperda* (JE Smith) in functional response experiments in laboratory arenas [10]. The theoretical maximum daily prey consumption rate, instantaneous attack rate, and handling time estimates indicated that *H*. *axyridis* (rather than *O*. *sauteri*) was a voracious predator of *S*. *frugiperda* eggs and young larvae [10].

Several articles tested the utilization of entomopathogens as natural enemies of crop pests. Seven species of entomopathogenic nematodes in genera *Heterorhabditis* and *Steinernema* were tested against late larval stages and pupae of *S*. *frugiperda* in the soil [11]. Three nematode species *Heterorhabditis indica* (Poinar), *Steinernema carpocapsae* (Weiser), and *Steinernema longicaudum* Shen & Wang were most virulent in soil column and pot bioassays [11]. Another article tested the pathogenicity of a novel *Metarhizium* fungus, i.e., *Metarhizium* sp. BCC 4849, on tetranychid spider mites (*Tetranychus truncatus* Ehara and *Eutetranychus africanus* Tucker) and several insect pests [12]. The mortality of *T*. *truncatus* exceeded 80% five days post-inoculation with this fungus. The fungus caused 92–99% mortality of cassava mealybug (*Phenacoccus manihoti* Matile-Ferrero), bean aphid (*Aphis craccivora* Koch), Oriental fruit fly (*Bactrocera dorsalis* Hendel), and sweet potato weevil (*Cylas formicarius* F.) within three-to-six days, post-inoculation [12].

*Metarhizium* were also tested for compatibility with a braconid parasitoid *Diachasmimorpha longicaudata* (Ashmead) to control a tephritid fruit fly *Anastrepha ludens* Loew [13]. *Metarhizium robertsii* strain V3-160 and *Metarhizium anisopliae* strain MAAP1 caused moderate levels of mortality in *D*. *longicaudata* adults. However, *M*. *robertsii* had no adverse effects on *D*. *longicaudata* larvae developing inside treated hosts, *A*. *ludens* [13]. In another compatibility study, entomopathogenic fungi *Beauveria bassiana* (two strains) and trichogrammatid parasitoids in genus *Trichogramma* were evaluated for combined utilization against the European pepper moth *Duponchelia fovealis* (Zeller), family Crambidae [14]. Both *B*. *bassiana* strains had minimal adverse effects on *Trichogramma* species. The authors suggested that *Beauveria* and *Trichogramma* could be combined to control *D*. *fovealis* under natural conditions [14].

Trichogrammatids have a long history of utilization to manage the egg stage of lepidopteran pests, and articles in this collection also tested the capacity of *Trichogramma* species to control a gelechiid *T*. *absoluta* [15], a crambid *Ostrinia furnacalis* Güenée [16], and a noctuid *Helicoverpa armigera* (Hübner) [17]. Nine European *Trichogramma* species were evaluated as candidates for biological control of *T*. *absoluta*, which was introduced to Europe in 2006. Results indicated that three species, *Trichogramma nerudai* Pintureau & Gerding, *Trichogramma pintoi* Voegele, and *Trichogramma cacoeciae* Marchal, were just as effective as commercially available *Trichogramma achaeae* Nagaraja & Nagarkatti in laboratory bioassays [15]. In another article, research demonstrated that *Trichogramma ostriniae* Pang & Chen and *Trichogramma dendrolimi* Matsumura were capable of parasitizing *O. furnacalis* eggs [16]. *Trichogramma ostriniae* attacked young and old host eggs, whereas *T*. *dendrolimi* preferentially attacked young host eggs. Since host egg age had no effect on *T*. *ostriniae* parasitism rate, authors suggested that *T*. *ostriniae* would be the best candidate for biological control of *O*. *furnacalis* [16]. One final article tested the suitability of *H*. *armigera* as a rearing host for *Trichogramma euproctidis* (Girault) [17]. Authors determined that host egg age was important; young rather than old *H*. *armigera* eggs were best for optimal development and reproduction of *T*. *euproctidis* in the laboratory [17].

In conclusion, this editorial summarizes research collected on a topic of considerable interest and importance: natural enemies and biological control of plant pests. This topical collection demonstrates that researchers continue to search for new methods and techniques to manage natural enemies, singly or combined with other species, with the ultimate goal of integrating them into pest management systems that rely less and less on harmful pesticides. Future research should continue to discover new and improved technologies to mass produce and release natural enemies, manipulate their densities in agroecosystems, and conserve their populations within agricultural landscapes throughout the world.

## Data Availability

Not applicable.
